# Oxytocin curbs calorie intake via food-specific increases in the activity of brain areas that process reward and establish cognitive control

**DOI:** 10.1038/s41598-018-20963-4

**Published:** 2018-02-09

**Authors:** Maartje S. Spetter, Gordon B. Feld, Matthias Thienel, Hubert Preissl, Maike A. Hege, Manfred Hallschmid

**Affiliations:** 1 Institute of Medical Psychology and Behavioural Neurobiology, University of Tübingen, Tübingen, Germany; 2 School of Psychology, University of Birmingham, Edgbaston, Birmingham UK; 3 Institute of Behavioural Neuroscience, Department of Experimental Psychology, Division of Psychology and Language Science, University College London, London, UK; 4 Department of Internal Medicine, Division of Endocrinology, Diabetology, Angiology, Nephrology and Clinical Chemistry, University of Tübingen, Tübingen, Germany; 5 Department of Pharmacy and Biochemistry, Institute of Pharmaceutical Sciences, University of Tübingen, Tübingen, Germany; 6 Interfaculty Centre for Pharmacogenomics and Pharma Research, University of Tübingen, Tübingen, Germany; 7 Institute for Diabetes and Obesity, Helmholtz Diabetes Center, Helmholtz Center Munich, German Research Center for Environmental Health, Neuherberg, Germany; 8 German Center for Diabetes Research (DZD), Tübingen, Germany; 9 Institute for Diabetes Research and Metabolic Diseases of the Helmholtz Center Munich at the University of Tübingen, Tübingen, Germany

## Abstract

The hypothalamic neurohormone oxytocin decreases food intake via largely unexplored mechanisms. We investigated the central nervous mediation of oxytocin’s hypophagic effect in comparison to its impact on the processing of generalized rewards. Fifteen fasted normal-weight, young men received intranasal oxytocin (24 IU) or placebo before functional magnetic resonance imaging (fMRI) measurements of brain activity during exposure to food stimuli and a monetary incentive delay task (MID). Subsequently, ad-libitum breakfast intake was assessed. Oxytocin compared to placebo increased activity in the ventromedial prefrontal cortex, supplementary motor area, anterior cingulate, and ventrolateral prefrontal cortices in response to high- vs. low-calorie food images in the fasted state, and reduced calorie intake by 12%. During anticipation of monetary rewards, oxytocin compared to placebo augmented striatal, orbitofrontal and insular activity without altering MID performance. We conclude that during the anticipation of generalized rewards, oxytocin stimulates dopaminergic reward-processing circuits. In contrast, oxytocin restrains food intake by enhancing the activity of brain regions that exert cognitive control, while concomitantly increasing the activity of structures that process food reward value. This pattern points towards a specific role of oxytocin in the regulation of eating behaviour in humans that might be of relevance for potential clinical applications.

## Introduction

Oxytocin is produced in the paraventricular nucleus of the hypothalamus and released into the circulation via the posterior pituitary; in addition it is secreted directly into limbic, hind- and midbrain regions^1^. Oxytocin modulates psychosocial function and affective processing like social bonding, emotion regulation, childbirth, and sexual behaviour^2–4^ (for review see ref.^1^) by influencing the interplay between amygdalar pathways, key regulators of emotional behaviour^5^, and prefrontal cortical networks^6^. Effects on the brain’s reward circuitry, in particular on dopaminergic signalling, likely contribute to the psychosocial impact of the hormone^7,8^. Research in animals and humans consistently indicates that oxytocin also acts as an anorexigenic factor in the control of food intake^9–13^. Lesions of oxytocin-expressing hypothalamic nuclei result in increased food intake and body weight^14^, whereas the administration of oxytocin inhibits eating behaviour^12,15^. Of particular interest in the clinical context, oxytocin reduces food intake also in animals with diet-induced obesity^16,17^. More recently, the peptide has been demonstrated to improve peripheral glucose homeostasis in healthy humans^18^.

**Figure 1:**
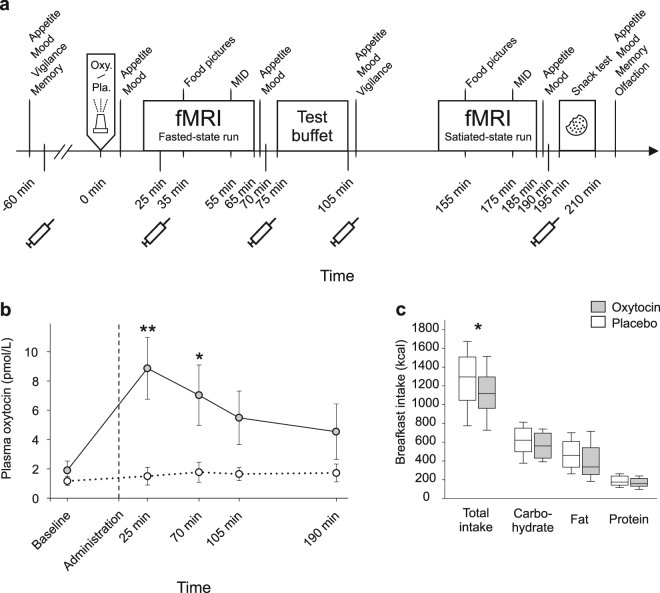
Experimental procedure, plasma oxytocin concentrations and breakfast intake. (**a**) Following baseline assessments of food-related and psychological variables and blood sampling, healthy young men were intranasally administered oxytocin (24 IU) and placebo, respectively, around 09.00 h (t = 0; spray symbol). After substance administration, participants were placed in the fMRI scanner to first undergo several technical scans followed by the food picture (35 min post-adminsitration) and the monetary incentive delay (MID) task (55 min), with an additional blood sampling in between. Seventy-five min after administration, subjects were allowed to eat ad libitum from a free-choice test buffet for 30 min, and the fMRI scans were repeated in the postprandial state. Around 85 min after termination of the buffet, snack intake was measured under the pretext of a taste-rating task, olfactory function was tested and appetite as well as short-term memory were assessed. (**b**) Mean plasma oxytocin concentrations (±SEM) assessed before and after intranasal administration (upright dotted line) of oxytocin (24 IU; grey dots and solid lines) and placebo (vehicle; white dots and dotted lines). (**c**) Calorie consumption from a test breakfast offered 75–105 min post-administration. N = 14–15; **p < 0.01, *p < 0.05 for comparisons between conditions (pairwise t-tests).

Studies in humans indicate that oxytocin administered to the brain via the intranasal route curbs the consumption of palatable snacks in normal-weight individuals^11^ and acutely reduces breakfast intake in normal-, but also overweight subjects^9^. The hypophagic effect of oxytocin may even be more pronounced in obese than normal-weight men^13^, and intranasal oxytocin administration for eight weeks has been found to reduce body weight in obese subjects^19^. The neuronal mechanisms underlying oxytocin’s restraining effect on ingestive behaviour are only poorly understood. In animals, food intake increases oxytocin secretion from the pituitary^20^, and the peptide acts as a downstream mediator of the anorexigenic effect of the adipocyte leptin by sensitizing caudal brain stem nuclei to satiety signals like cholecystokinin^21–23^. Still, eating behaviour does not merely depend on food deprivation and consumption leading to hunger and, respectively, satiety, but implies a strong reward-related component^24^. Respective experiments in humans available so far^9,11,13^ indeed suggest that oxytocin decreases food intake in part by acting on reward-processing neuronal circuits. However, it is unclear whether the contribution of oxytocin to the regulation of human eating behaviour is established via food-specific effects on distinct brain networks or, rather, is a by-product of oxytocin’s involvement in general reward processing; our study was performed to address this question. In short, healthy, fasted men received intranasal oxytocin (24 IU) or placebo before their brain activity was assessed via fMRI scans while they looked at food and non-food pictures and performed the monetary incentive delay (MID) task, which assesses responses to monetary rewards (fasted-state runs). Subsequently, subjects ate from an ad-libitum breakfast buffet and the food picture and MID tasks were repeated in the scanner for exploratory purposes (satiated-state runs). Finally, casual snack intake was covertly assessed (see Fig. 1a for experimental procedure). We expected to identify eating-related brain mechanisms that mediate the hypophagic effect of oxytocin, but differ from those that establish oxytocin’s role in the processing of generalized (monetary) rewards.

## Results

### Plasma oxytocin concentrations, food intake, appetite and auxiliary measures

Plasma oxytocin concentrations increased after intranasal administration of the peptide (F(1,116) = 8.91, *p* < 0.001 for treatment × time), reaching peak values of 8.87 ± 2.11 pmol/L 25 min after administration (Fig. 1b). Oxytocin compared to placebo curbed calorie intake by around 150 kcal or 12% during breakfast (F(1,13) = 8.22, *p* = 0.013; Fig. 1c). This effect was reflected by trend-wise decreases in the consumption of fat (F(1,13) = 4.41, *p* = 0.056), protein (F(1,13) = 4.16, *p* = 0.062) and carbohydrates (F(1,13) = 3.60, *p* = 0.068), but did not differ between macronutrients (*p* = 0.30 for treatment × macronutrient; Table 1). Oxytocin neither induced a shift in the intake of different food types, i.e., sweet, savoury and neutral items (F(1,69) = 0.02, *p* = 0.98 for treatment × type). Snack intake measured 10 min after the satiated-state fMRI scans, i.e., around 3 hours after substance administration, did not differ between conditions (all *p* > 0.46; Table 1). Calorie intake collapsed across breakfast and snacks was likewise reduced by oxytocin compared to placebo (1294 ± 112 vs. 1438 ± 113 kcal, F(1,14) = 5.51, *p* = 0.034). The effects of oxytocin on calorie intake remained unchanged when corrected for body weight.

**Table 1:** Calorie intake (kcal) during the breakfast buffet and the snack test.

Eating assessment		Placebo	Oxytocin	*p* value
**Breakfast**	**Total**	**1271 ± 77**	**1121 ± 69**	**0.013**
*Macronutrient*	Carbohydrate	612 ± 40	557 ± 35	0.068
	Fat	473 ± 43	398 ± 49	0.056
	Protein	186 ± 13	166 ± 14	0.062
**Snack test**	**Total**	**167 ± 18**	**172 ± 24**	**0.78**
*Snack type*	Chocolate cookies	90 ± 21	93 ± 21	0.87
	Rice waffles	23 ± 7	23 ± 6	1.00
	Salty crackers	60 ± 16	56 ± 13	0.73

Complete lists of breakfast ingredients and nutritive values of breakfast and snacks can be found in ref.^13^. *p* values are derived from mixed ANOVA. N = 14.

Rated hunger declined after breakfast by around 85% across conditions, without differences between conditions (*p* = 0.43). All other measures of desire for food including the food control questionnaire-state were not affected by oxytocin (all *p* > 0.5). Auxiliary measures of mood, vigilance, working memory, and olfactory function were likewise comparable between conditions (see Supplementary Information). Participants could not differentiate between oxytocin and placebo treatment (*p* > 0.44, chi-squared test).

### Food picture task

#### Food vs. non-food stimuli

In the fasted-state run, food pictures compared to non-food pictures evoked stronger responses in bilateral insula, lateral orbitofrontal cortex (OFC), ventromedial prefrontal cortex (vmPFC), fusiform gyrus, frontal gyrus, parahippocampus, and putamen, without modulatory effects of oxytocin. Respective effects in the satiated state focused on the insula (all whole-brain analyses, *p* < 0.05 family-wise error (FWE)-corrected; see Table 2 for the main food-related fMRI results).

**Table 2:** fMRI-assessed brain responses during food picture presentation.

Brain region	Peak voxel coordinates			Z score	Cluster size^a^	*p* value (FWE-corrected)^b^
	x	y	z			
**Food vs. non-food pictures**						
*Fasted-state run*						
Fusiform	15	−85	−4	Inf	2291	0.001
Insula L	−36	−7	14	7.28	153	0.001
	−39	−4	2	6.67		
	−36	2	−10	6.03		
Insula R	36	−4	17	6.78	105	0.001
	39	−4	8	6.77		
	36	5	−13	6.30		
OFC L	−27	29	−13	6.27	19	0.001
Parahippocampus	21	−4	−22	5.79	12	0.001
Inferior frontal gyrus	−48	35	17	5.79	49	0.001
	30	32	−10	5.50		
vmPFC	51	41	17	6.49		
OFC R	27	35	−10	5.54	34	0.001
Putamen R	21	−10	−4	5.38	8	0.001
*Satiated-state run*						
Fusiform	15	−85	−4	Inf	1470	0.001
Insula L	−39	2	−10	6.44	92	0.001
	−36	−10	14	6.39		
Insula R	39	−4	8	6.28	78	0.001
	39	5	−10	5.67		
**High- vs. low-calorie food pictures**						
*Oxytocin vs. placebo—Fasted-state run*						
vmPFC L	−9	47	−7	4.57	170	0.001
	−6	56	14	3.59		
	3	47	−15	3.77		
SMA R	12	20	59	4.46	270	0.001
	−9	20	32	4.45		
	12	17	41	3.98		
ACC (ROI)	−6	26	29	4.38	32^c^	0.016
vlPFC L (ROI)	−54	11	11	4.06	22^c^	0.027
	−48	11	−1	3.71		
vlPFC R (ROI)	54	23	8	3.86	26^c^	0.034
*Oxytocin vs. placebo–Satiated-state run*	n.s.					

Results are FWE-corrected for multiple comparisons (*p* < 0.05). ^a^For cluster sizes of FWE-corrected clusters, main effects of food vs. non-food stimuli are thresholded at FWE-corrected level; oxytocin effects are based on a primary uncorrected threshold level of *p* < 0.001. ^b^*p* values are all FWE-corrected, for ^c^ROI analyses at peak level (*p* < 0.05 FWE-corrected). Coordinates are in Montreal Neurological Institute (MNI) space; anatomy labels are given according to the AAL brain atlas. L, left hemisphere, R, right hemisphere, OFC, orbitofrontal cortex, vmPFC, ventromedial prefrontal cortex, SMA, supplementary motor area, ACC, anterior cingulate cortex, vlPFC, ventrolateral prefrontal cortex. N = 15.

#### Oxytocin effect on high- vs. low-calorie food stimuli

**Figure 2:**
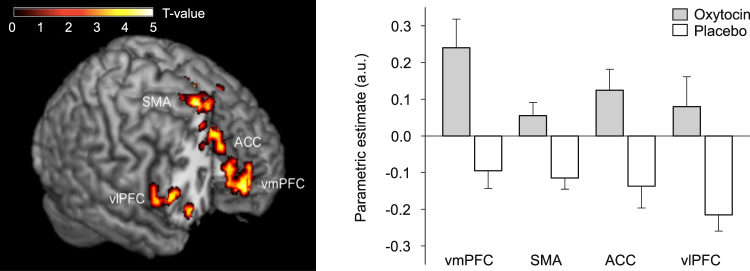
Oxytocin effects on neuronal activity during food picture presentation. Oxytocin-induced relative increases in neuronal activity (compared to placebo) in response to high- vs. low-calorie food pictures presented in the fasted state, and respective parametric estimates (±SEM). vmPFC, ventral medial prefrontal cortex, SMA, supplementary motor area, ACC, anterior cingulate cortex, vlPFC, ventrolateral prefrontal cortex; threshold of *p* < 0.001, uncorrected for multiple comparisons k > 25. N = 15.

Analyses distinguishing between the responses to high- and low-calorie food pictures indicated that when subjects were fasted, oxytocin compared to placebo increased activity in response to high- vs. low-calorie food items in the vmPFC, supplementary motor area (SMA), anterior cingulate cortex (ACC), and bilateral ventrolateral prefrontal cortex (vlPFC; all region of interest (ROI) analyses, *p* < 0.05, FWE-corrected at cluster and peak level; Fig. 2 and Table 2). Analyses of the satiated-state runs indicated that these effects were restricted to the fasted-state, i.e., pre-breakfast measurements.

Comparisons of the effects of oxytocin on brain activation in response to high- vs. low-calorie food stimuli and breakfast intake yielded a positive correlation between the oxytocin-induced increase in activation of the right vlPFC and the respective decrease in calorie intake from sweet breakfast ingredients (r = 0.57, *p* = 0.034). Corresponding, albeit only trend-wise results emerged for the relationship between increases in left vlPFC activation and decreases in calorie intake from savoury breakfast items (r = 0.50, *p* = 0.069) as well as for the respective relationships between SMA and, respectively, ACC activation and total breakfast intake when measured in grams (r = 0.49, *p* = 0.076; r = 0.46, *p* = 0.099), but not calories (n.s.). We also found a positive correlation of oxytocin plasma peak concentrations and, respectively, area under the curve (AUC) values in both conditions with activation in the ACC (r = 0.39, *p* ≤ 0.05; r = 0.48, *p* = 0.01) and the vlPFC (r = 0.43, *p* = 0.03; r = 0.51, *p* ≤ 0.01). Note that these correlative analyses were performed in an exploratory fashion.

### MID task

#### Performance

In each trial of the MID task, the participant first saw a cue symbol announcing potential gains, losses, or no monetary outcome. The target stimulus appeared within a variable delay after the cue, and subjects, in dependence on the specific cue stimulus, had to press a button to win money or to avoid losing money, or had to abstain from responding. They were notified about their gains and losses after each trial.

In the fasted state, hit rate, i.e., proportion of successful button presses during target presentation (mean 63 ± 0.7%), and reaction times for hits (mean 197 ± 3.0 ms) did not differ between conditions (*p* > 0.19 for all comparisons). Reaction times were lower for responses to cues announcing comparatively higher rewards (F(1,180) = 6.00, *p* < 0.001). Comparable results were found in the satiated state (hit rate, 64 ± 1%; reaction times for hits, 185 ± 2.9 ms, all *p* > 0.30; F(1,180) = 13.5, *p* < 0.001 for comparisons of responses to cues announcing higher vs. lower rewards).

#### Effect of anticipation

Anticipating the execution of a response in comparison to no response (possibility to win or lose compared to neutral cue) in the fasted state was associated with increased activation of motor cortex, bilateral striatum, precentral gyrus, insula, midbrain, and OFC (whole-brain level, all *p* < 0.05 FWE-corrected; see Table 3 for main MID-related results). This effect was increased in response to high-value (5 Euro) cues (bilateral striatum and motor cortex; both *p* < 0.05 FWE-corrected). A comparable pattern was observed in the satiated state (see Table 3). We did not observe interactions between treatment and the incentive value of the cues.

**Table 3:** fMRI-assessed brain responses during MID task performance.

Brain region	Peak voxel coordinates			Z score	Cluster size^a^	*p* value (FWE-corrected)^b^
	x	y	z			
**Anticipating response vs. no-response**						
*Fasted-state run*						
Motor cortex	−6	−1	56	Inf	2120	0.001
Striatum L	−21	5	5	Inf		
Precentral gyrus	−36	−16	52	Inf		
Striatum R	21	8	−1	Inf	551	0.001
Insula	36	20	8	7.81		
Midbrain	6	−25	−10	7.78		
OFC	21	41	−13	5.72	12	0.002
*Satiated-state run*						
Motor cortex	−6	−1	56	Inf	4206	0.001
Striatum	−21	5	5	Inf		
Precentral gyrus	−36	−16	53	Inf		
vmPFC	36	38	29	6.07	41	0.001
	−39	35	29	5.96		
*Oxytocin vs. placebo—Fasted-state run*						
Frontal inferior operculum	−48	17	17	6.96	16	0.001
OFC	−39	35	−4	5.81	22	0.001
	36	35	−7	4.93	5	0.008
Insula (ROI)	39	17	11	4.69	6^c^	0.001
Striatum (ROI)	30	8	−7	4.41	16^c^	0.004
	9	14	−7	4.28	13^c^	0.007
	−24	2	−7	4.24	16^c^	0.008
*Oxytocin vs. placebo—Satiated-state run*						
Hypothalamus (ROI)	−3	−10	−1	6.05	9^c^	0.001
**Feedback (win; hit vs. miss)**						
*Fasted-state run*						
Medial OFC	−6	50	−7	5.25	16	0.001
Caudate	21	−4	26	5.15	9	0.001
	18	8	23	4.93		
**Feedback (no loss; hit vs. miss)**						
*Fasted-state run*						
Striatum (putamen)	27	5	8	5.01	14	0.001
	24	8	17	4.92		

Results are FWE-corrected for multiple comparisons (*p* < 0.05). ^a^All cluster sizes are thresholded at FWE-corrected level (*p* < 0.05). ^b^*p* values are all FWE-corrected, for ^c^ROI analyses at peak level (*p* < 0.05 FWE-corrected). Voxel coordinates are in Montreal Neurological Institute (MNI) space; anatomy labels are given according to the AAL brain atlas. OFC, orbitofrontal cortex, vmPFC, ventromedial prefrontal cortex. Note that differences between conditions in satiated-state hypothalamic activity vanished when corrected for respective differences in preceding breakfast intake. N = 15.

#### Oxytocin effect during anticipation

**Figure 3:**
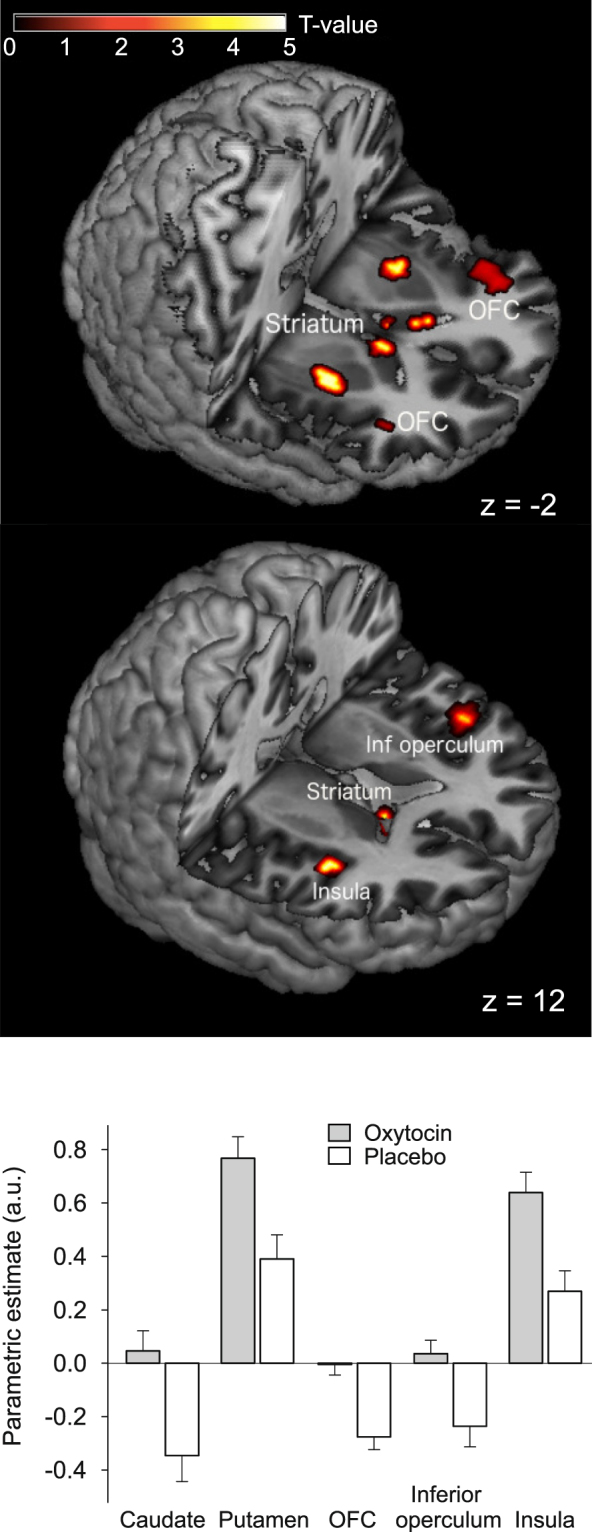
Oxytocin effects on neuronal activity during anticipation of monetary rewards and losses. Oxytocin-induced relative increases in neuronal activity (compared to placebo) in anticipation of rewards and losses during the MID task measured in the fasted-state run, and respective parametric estimates (±SEM). OFC, orbitofrontal cortex, inf. operculum, inferior operculum; threshold of *p* < 0.001, uncorrected for multiple comparisons for left caudate, left and right putamen, and insula k > 25. N = 15.

In the fasted-state run, oxytocin compared to placebo elicited increased activation in the OFC, bilateral putamen, left caudate, frontal inferior operculum, and insula (all *p* < 0.05 FWE-corrected) during anticipation of rewards and losses (Fig. 3 and Table 3). In the satiated-state run, activation of the hypothalamus at first appeared to be increased by oxytocin (*p* < 0.05 FWE-corrected), but this effect was not significant when calorie intake during preceding breakfast was included as a covariate. We did not observe any differences between conditions in the neuronal activation in response to the possibility of win/high gain vs. no win/low gain. Further explorative analyses indicated positive correlations of oxytocin plasma peak concentrations and, respectively, AUC values with activation of OFC (r = 0.44, *p* = 0.02; r = 0.45, *p* = 0.02) and putamen (r = 0.61, *p* ≤ 0.01; r = 0.58, *p* = 0.02) when analysed across conditions.

#### Effect of feedback

In the fasted-state run, feedback on wins in comparison to misses was associated with increased activation of the medial OFC and caudate (both *p* < 0.05 FWE-corrected). Striatal activation (putamen) increased (*p* < 0.05 FWE-corrected) when subjects avoided losing money (Table 3). No differences between conditions were observed with regard to feedback effects.

## Discussion

Our study in healthy men indicates that the brain mechanisms underlying the inhibitory effect of oxytocin on food intake can be differentiated from mediators of oxytocin-induced changes in the processing of generalized, monetary rewards. Intranasal oxytocin administration to food-deprived subjects altered fMRI-assessed neuronal activation patterns in food intake-regulatory pathways in response to food stimuli. Activity in the vmPFC as well as in SMA, vlPFC, and ACC was enhanced by oxytocin during exposure to high- vs. low-calorie visual food stimuli, indicating that the neuropeptide on the one hand increases the activity of networks that process the reward value of food and, on the other hand, enhances the function of structures that mediate cognitive control of consummatory behaviour. Fittingly, oxytocin compared to placebo also decreased subsequent ad-libitum breakfast intake by around 12%. Oxytocin did not affect behavioural performance on the MID task, although orbitofrontal, insular and striatal activation was enhanced by oxytocin during the anticipation of monetary rewards or losses. Our results indicate that the restraining effect of oxytocin on calorie intake found in previous^9,11,13^ and corroborated in the current study may not be a mere by-product of changes in general reward processing, but rather be driven by specific alterations in central nervous networks which process higher cognitive processes related to the acute control of food intake.

When our participants viewed high- as compared to low-calorie food stimuli in the fasted state, oxytocin in comparison to placebo increased neuronal responses in vmPFC, SMA, vlPFC, and ACC. Activity of the vmPFC is known to be positively correlated with the reward value of food stimuli^25,26^, suggesting that the inherent rewarding value of the high-calorie food items and associated reward expectancy were enhanced by oxytocin. This assumption is in line with previous studies showing that intranasal oxytocin increases connectivity between vmPFC/OFC, ACC and thalamus^6^. Moreover, frontal structures contribute to emotional evaluation and modulate activation of the amygdala^27^, a key emotion-processing brain structure that responds to oxytocin^1,5^. Importantly, oxytocin also increased activation of the SMA which receives input from the striatum and is associated with inhibitory processes in the regulation of the desire for palatable food^28^ and the success of self-regulation^29^. The oxytocin-induced increase in vlPFC activation supports the conclusion of an oxytocin-induced improvement in inhibitory control^28^, not least because this structure is engaged in focusing on the long-term cost of consuming unhealthy food^30^. Increased activity of the ACC, which contributes to emotional regulation during cognitive reappraisal^31^ and self-regulation of food intake^32^, may add to oxytocin-induced improvements of cognitive control capacities. This assumption is buttressed by the reduction in ad-libitum breakfast intake by around 150 kcal or 12% of the amount ingested in the placebo condition. Our correlational findings moreover yielded tentative evidence for a functional relationship between the stimulating effect of oxytocin on vlPFC, SMA and ACC activation and its inhibitory effect on food intake. Fittingly, a trend towards reduced food craving along with signs of increased neuronal activity in prefrontal regions was found in women after oxytocin administration^33^. Oxytocin-induced increases in the activity of areas that mediate inhibitory control might override the stimulating effect of the neuropeptide on reward value representation that is indicated by increased vmPFC activity, and attenuate actual food intake. This interpretation converges with our finding that actual desire for food did not differ between conditions, but should be further substantiated in fine-grained investigations into oxytocin’s role in food-related cognitive control.

Acute oxytocin-induced decreases in energy intake have been found in normal- and overweight humans^9,11,13^. Whereas our previous results suggested that the hypophagic effect of oxytocin in normal-weight humans focuses on reward-driven snacking in the satiated state^11^, in the present study oxytocin reduced hunger-driven calorie consumption during breakfast but did not affect snack intake, which took place somewhat later than in the aforementioned studies. Subtle differences in experimental timing and set-up may underlie these discrepancies that underline the need for further studies of the behavioural specifics of oxytocin’s anorexigenic impact. They will be of particular relevance when potential clinical uses are considered. In line with previous experiments^11,13^, self-rated hunger was not affected by oxytocin. Rather than influencing the motivation to eat, oxytocin therefore might contribute to the termination of meals by enhancing inhibitory control mechanisms. We could not confirm recent reports of oxytocin-induced decreases in hypothalamic activation in response to food cues^34^. Food intake in animals triggers c-Fos protein expression in hypothalamic oxytocin neurons^35^ and increases plasma oxytocin concentrations^20^. In our study, oxytocin appeared to increase hypothalamic activity during MID reward/loss anticipation in the satiated state, but this effect vanished after correction for preceding breakfast intake. It is to note that the fixed temporal sequence of fasted- and satiated fMRI runs in the present experiments may have lead to habituation effects, which could explain the scarcity of oxytocin-induced postprandial changes in brain activity and renders respective conclusions preliminary.

Performance on the MID task was not affected by oxytocin, indicating that oxytocin exerts no or only mild acute effects on the behavioural consequences of processing generalized rewarding stimuli. Oxytocin in comparison to placebo increased activation of caudate and putamen during the anticipation of rewards and losses, key areas of the dopaminergic reward network, and induced a trendwise increase in insular activity. These results accord with oxytocin effects in patients with posttraumatic stress disorder and controls who performed a social version of the MID task^36^, which points to a broad impact of oxytocin on reward-processing structures. Oxytocin-induced enhancements of striatal and insular responses during the anticipation of monetary rewards moreover concur with improved responses in the mesolimbic dopaminergic system found in studies on social reward and punishment^8,37,38^. This pattern suggests that the strong psychosocial impact of oxytocin is mediated to a large extent by its effect on the brain’s reward circuitry, including limbic prefrontal areas^39^.

In sum, we show that intranasal oxytocin administration to fasted normal-weight subjects exerts stimulatory effects on fronto-cortical brain regions that encode the reward value of a stimulus as well as on connected areas that establish inhibitory control of behavioural responses, which in concert may mediate the observed reduction in food intake. During the anticipation of monetary, generalized rewards, oxytocin increases activity in left caudate and bilateral putamen without immediate behavioural consequences. In some contrast to its primarily reward-related effects on psychosocial behaviour, oxytocin appears to limit food intake in humans by enhancing the activity of fronto-cortical brain areas that establish cognitive control processes, thereby overriding the hedonic drive to eat. In light of the promising results of proof-of-concept experiments in subjects with elevated body weight^9,13^, this mechanism of action may still be functional in obesity, which has been reported to be associated with alterations in circulating oxytocin concentrations^40–42^, and open up new avenues to normalize eating behaviour in the clinical context^43,44^.

## Methods

### Subjects

Twenty healthy male non-smokers participated in the study (mean age ± SEM, 25.7 ± 2.6 years, BMI, 22.7 ± 1.3 kg/m^2^) after a screening session including clinical examination. Exclusion criteria included eating disorders, excessive alcohol consumption, any diseases, medication, and contraindications for MRI. The final sample consisted of fifteen participants because one subject withdrew from participation after one session, and data sets of four subjects had to be omitted from fMRI analyses due to movement artefacts. One additional subject had to be omitted from the analyses of energy intake because of a technical failure (missing breakfast ingredients in one session). Participants gave written informed consent to the study that confirmed to the Declaration of Helsinki and was approved by the Ethics Committee of the Medical Faculty of the University of Tübingen. Participants received 100 Euro upon completion of the study.

### Study design and procedure

Experiments were carried out in a double-blind, crossover, within-subject design. Subjects participated in two sessions (oxytocin and placebo) on two separate days at least 14 days apart. The order of conditions was balanced across participants, who were kept unaware of hypothesized treatment effects on food intake. Participants were instructed to abstain from food and caffeinated/alcoholic beverages after 20.00 h on the day preceding each session.

Upon arrival of the subject between 07.00 and 08.00 h in the morning, blood was sampled for baseline assessments of oxytocin via a venous cannula placed in the subject’s arm, and subjects underwent tests characterizing appetite, food preferences, vigilance, memory function and mood. After a practice-run of the MID task, subjects were intranasally administered oxytocin (24 IU in 0.6 ml, Syntocinon, Defiante Farmacêutica, Funchal Madeira, Portugal) or placebo (vehicle) via six 0.1-ml puffs (three per nostril) at 30-s intervals; this dose of intranasal oxytocin is known to increase blood as well as cerebrospinal fluid concentrations of the peptide^45^. Thirty-five min after substance administration, the 16-min food picture and the 13-min MID tasks were performed in the scanner (fasted-state runs). Seventy-five min after administration, subjects were presented with an ad-libitum breakfast buffet and subsequently again underwent the appetite/food/vigilance/mood test set. Thereafter, the food picture and MID tasks were repeated in the scanner (satiated-state runs) and an anatomical scan was performed. Eventually, casual snack intake was covertly assessed and working memory and olfactory performance were tested. Appetite and mood questionnaires were repeatedly filled in during sessions.

#### Food picture task

Food stimuli were standardized images of sweet and savoury food items (e.g., apples and pretzel sticks) and non-food items matched for size and shape (office supplies; e.g., pencils) provided by Charbonnier and colleagues^46^. Ninety food (45 low-calorie and 45 high-calorie) and 45 non-food pictures were displayed in randomized order on a screen through a computer interface using Presentation (Neurobehavioural Systems Inc., Berkeley, CA). Pictures were presented for 3 s each with a variable inter-stimulus interval of 1–1.2 s in a jittered, pseudo-random sequence. To maintain attention, subjects intermittently (after ten picture stimuli on average) had to indicate via button press within 3 s if the most recent picture displayed a food or non-food item.

#### Monetary incentive delay task (MID)

The MID task adopted from Knutson and co-workers^47^ consisted of 25 trials in the practice run and 90 trials in the scanner runs. In each trial, the participant first saw a cue symbol (circle, square or triangle) for 250 ms that announced a potential gain (circle), potential loss (square), or no monetary outcome (triangle). The number of lines in each symbol indicated how much was at stake (between € 0 and € 5). The target stimulus (solid white square) appeared within a variable delay of 2–2.5 s after the cue, and subjects had to press a button to win money (in response to the circle cue) or to avoid losing money (square cue), or had to abstain from responding (triangle cue). Feedback (1.65 s) provided immediately after the button press notified participants of whether they had won or lost money during that trial and indicated their cumulative total at that point. Difficulty (the time given to respond to the target) was individually titrated according to performance at the practice run (ranging from 150–250 ms; see ref.^48^) to approximate a 66% hit rate. The different trial types were pseudo-randomly ordered within each session. Subjects were informed that they would earn the monetary reward in addition to their reimbursement.

#### Breakfast and snack intake

Seventy-five min after substance administration, subjects were offered an ad-libitum free-choice breakfast buffet comprising a variety of food choices^13^ from which they could eat undisturbed in a separate room for 30 min. They were told that breakfast was scheduled as a break between scan sessions, but were not aware that intake was measured by weighing buffet components before and after. Ninety min after breakfast, reward-related eating in the relative absence of hunger was assessed with a snack test validated in previous studies^11,49^ (see Supplementary Information).

#### Auxiliary measures and analyses of plasma oxytocin

Tests were performed at baseline and after substance administration (see Fig. 1a for experimental schedule) to assess oxytocin effects on appetite, food preferences, mood, vigilance, working memory, and olfactory function. Blood samples were repeatedly collected for the determination of plasma oxytocin concentrations. See Supplementary Information for details.

### Acquisition, processing and analysis of fMRI data

Scans were performed with a 3-Tesla PRISMA Siemens scanner with a 20-channel head coil. Functional images during the MID (367 volumes) and food picture task (475 volumes) were acquired with a single-shot echo-planar imaging (EPI) sequence with the following parameters: repetition time (TR = 2000 ms), adjusted flip angle (90°), echo time (TE = 30 ms), matrix size (64 × 64), and 30 slices (thickness = 3 mm), resulting in a voxel size of 3.3 × 3.3 × 3.0 mm^3^. The T_1_-weighted anatomical scan was acquired with a TR/TE of 61/8.4 ms, a flip angle of 30°, FOV of 288 × 175 mm, 175 axial slices, and a voxel size of 1 × 1 × 1 mm^3^.

Functional imaging data were analysed using SPM12 (Wellcome Department of Cognitive Neurology, London, UK) run with MATLAB 2013 (Mathworks Inc, Natick, MA) and the WFU Pickatlas-tool^50^ using standard procedures^51–54^. Functional image volumes were pre-processed, starting with slice timing and realignment of the images to the mean functional image. To allow for susceptibility by movement artefacts, unwarping of time series was performed. In addition, the high-resolution T1 image was co-registered to the mean image of the EPI series for each participant. Co-registered T1-weighted MR images were segmented for gray and white matter to compute spatial transformation parameters for normalization. The registered functional scans were normalized to a standard Montreal Neurological Institute (MNI) template. Normalized images were spatially smoothed with a 9 mm full-width half-maximum Gaussian kernel. A statistical parametric map was generated by fitting a boxcar function to each time series, convolved with the canonical hemodynamic response function. Data were high-pass filtered with a cut-off of 128 s. Scans that included head movements exceeding 3 mm in any direction during task performance were excluded from further analysis.

#### Within-subject analyses

Each subject underwent four scan sessions including measurements during the food picture and the MID tasks, i.e., oxytocin-fasted state, oxytocin-satiated state, placebo-fasted state, and placebo-satiated state. Condition-specific effects at each voxel were estimated using the general linear model. The response to events was modelled by a canonical hemodynamic response function^51^. For the food picture task, five conditions were modelled: viewing high-calorie food, low-calorie food, and non-food items, rest, and question. Responses to rest and question were neglected in further analyses. Two food-related neural activation contrast images were calculated: food vs. non-food (FvsNF) and high- vs. low-calorie food (HvsL). For the analysis of the MID task, 15 conditions were modelled: anticipation of potential gain of € 0, € 0.20, € 1 or € 5, anticipation of potential loss of € 0, € 0.20, € 1 or € 5, anticipation of no monetary outcome, rest, target, and feedback for win, no win, loss, no loss. In accordance with previous applications of the MID task^36,47,48^ contrast images were calculated for reward anticipation vs. neutral, loss anticipation vs. neutral, gain (€ 0.20, € 1 and € 5) vs. no gain (€ 0), and high gain € 5) vs. low gain € 0). For the analysis of feedback, four contrast images were calculated, win (reward/hit), no win (reward/miss), loss (loss avoidance/miss), and no loss (loss avoidance/hit; all vs. neutral). The motion correction parameters from the realignment procedure were added to all models as regressors to correct for motion-related variance.

#### Group-level analyses

Individual contrasts were entered into a repeated-measure ANCOVA including the factors ‘treatment’ (oxytocin or placebo) and ‘state’ (fasted or satiated). Order of conditions was introduced as an additional regressor. For the food picture task, two ANCOVAs were performed to compare neuronal activation in response to food vs. non-food items, and in response to high- vs. low-calorie items in dependence of treatment and state. Results of the MID task were analysed using respective repeated-measure ANCOVAs including the additional factor ‘outcome’ (gain or loss), resulting in a 2 × 2 × 2 design.

### Statistical analyses

Behavioural results and oxytocin concentrations were analysed using SPSS 22.0 (IBM Corp.) and are presented as means ± SEM. Analyses relied on a linear mixed effects model with a fixed treatment effect, time effect, and treatment × time/macronutrient/food type interaction as appropriate. Subjects were added as random variables. To adjust for baseline differences, baseline values were added as covariates in models with more than two measurements. Post-hoc, paired t-tests were performed (Bonferroni-corrected for multiple comparisons) to specify treatment effects. Statistical relationships between measurements of brain activation and behavioural as well as plasma oxytocin results were investigated in an exploratory fashion by calculating two-tailed Pearson correlations between neuronal activation (beta-values of significant task-specific brain activation clusters), peak and AUC values of plasma oxytocin, and food intake parameters. For breakfast-related analyses, the included measures were the differences between conditions in vmPFC, SMA, ACC, vlPFC (L) and vlPFC (R) activation, and in total breakfast intake (in kcal and g) as well as in the intake (in kcal) from sweet and savoury items. A *p* value ≤ 0.05 was considered significant.

In fMRI analyses, main effects and interactions were considered significant at cluster level at *p* < 0.05, FWE correction, using a primary uncorrected threshold of *p* < 0.001 (k > 10)^55^. In addition to whole-brain analyses, ROI analyses with a priori defined masks were performed. Predicted regions for oxytocin effects were considered significant at *p* < 0.05, FWE-corrected at cluster and peak level within regions of interest defined according to previous findings. For the food picture task, they covered prefrontal areas as indicated by Striepens and colleagues^33^, as well as the OFC, inferior frontal and fusiform gyrus and insula whose activity increases during the presentation of food pictures^54,56^. With regard to the MID task, Nawijn and colleagues^36^ reported on oxytocin-induced changes in insula and striatum activation during the MID task; prefrontal areas and amygdala were found by Knutson and colleagues to be sensitive to MID performance^57^. For display, all maps are thresholded at *p* < 0.001 uncorrected with a cluster criterion of 25 voxels.

### Data availability

The datasets generated during the current study are available from the corresponding author on reasonable request.

## Electronic supplementary material

Supplementary information
