# Supplementary material for: Oxytocin curbs calorie intake via food-specific increases in the activity of brain areas that process reward and establish cognitive control
Source: Sci Rep. 2018 Feb 9;8:2736. doi: 10.1038/s41598-018-20963-4 (PMC5807408; doi:10.1038/s41598-018-20963-4)
Supplement: Supplementary file 1 — Supplementary information [file 41598_2018_20963_MOESM1_ESM.doc]

**Supplementary Information**

*Oxytocin curbs calorie intake via food-specific increases in the activity of brain areas that process reward and establish cognitive control*

Maartje S. Spetter, Gordon B. Feld, Matthias Thienel, Hubert Preissl, Maike A. Hege, Manfred Hallschmid

*Snack intake.* In the snack test for the assessment of reward-related eating that took place 90 min after breakfast, subjects were presented three types of snacks of different taste but comparable calorie content and macronutrient composition, each on a separate plate, and labeled snack A, B, and C, respectively. The three types were, “TUC Cracker Classic” (salty taste; Griesson-de Beukelaer, Polch, Germany), “Rice Waffles” (bland taste; Continental Bakeries B.V., Dordrecht, The Netherlands), and “Double Chocolate Cookies” (sweet taste; EDEKA, Hamburg, Germany). In addition, a glass of water was provided. The participant was instructed to taste and rate each type of snack on a visual analog scale (VAS) anchored by 0 (not at all) and 100 mm (very palatable/sweet/salty). The importance of giving accurate ratings was emphasized and subjects were informed that during and after completion of the rating task they could eat as many snacks as they liked because any remaining snacks would be discarded, and were left alone for 10 min. Snack intake was covertly measured by weighing the snacks before and after the test.

*Auxiliary measures.* Appetite, food preferences, mood, vigilance, verbal working memory and olfactory function were assessed as follows. Subjects rated hunger, fullness, thirst, desire to eat in general, desire to eat something sweet or savory, nausea and anxiety on 100-mm VAS. They also filled in the food control questionnaire-state (FCQ-S1) Mood was assessed with a questionnaire containing 5-point scales covering the categories good/bad mood, alertness/sleepiness, and calmness/agitation^2^.

In the vigilance task^3^ a dot of 10 mm diameter appeared every 2, 4, 6, 8 or 10 seconds in the left or right field (randomly selected) of a computer screen and subjects were required to press corresponding left and right keys as fast as possible. Subjects received immediate feedback in the form of the reaction time (in ms) after a correct response or the signal ‘Wrong!’ after an incorrect response. The task included 40 trials (20 per screen half) and lasted approximately 5 min. For each 5-min task, mean reaction time, error rate and the number of lapses were registered.

Verbal working memory was assessed via the Digit Span subtest of the Hamburg-Wechsler Adult Intelligence Scale-Revised 1991^4^. In this test, up to nine digits were read to the subject at a rate of one digit per second, e.g. five-eight-two. The subject repeated the digits chronologically (forward test) or in reverse order (backward test). Starting with three digits, their length gradually increased until the subject failed to repeat the digits correctly twice in a row. Correct responses after one presentation equaled two points, and correct answers after two presentations equaled one point. The total performance score is the sum of the forward and backward components (maximum score: 28 points). At the end of the session, olfactory function was tested with the validated Sniffin’ Sticks commercial test kit (Burghart Elektro- und Feinmechanik GmbH, Wedel, Germany) that allows for the separate characterization of the three dimensions of olfactory threshold, discrimination, and identification^5^.

*Analyses of plasma oxytocin.* Blood samples were collected at baseline, i.e., before intranasal administration of oxytocin or placebo and at 25, 70, 105, and 190 min thereafter in tubes that contained aprotinine (370 kIU/ml; Roth GmbH, Karlsruhe, Germany). Samples were centrifuged and supernatants stored at -80°C. Plasma oxytocin concentrations were measured by radioimmunoassay after extraction by the Riagnosis Company (Regensburg, Germany), with a sensitivity in the 0.1 pg/sample range and intra- and interassay variabilities of < 10%.

**Supplementary Data**

*Auxiliary measures.* Mood and related control ratings were generally unaffected by oxytocin administration. Reaction times and error rates in the vigilance task as well as working memory performance remained stable across the experimental period (all *p* > 0.18) and did not differ between conditions (all *p* > 0.30). In the olfactory task at the end of experiments, we did not find indicators of oxytocin effects on olfactory discrimination (*p* > 0.99), identification (*p* > 0.55) and perception threshold (*p* > 0.15).

**Supplementary References**

1. Cepeda-Benito, A. *et al.* The development and validation of Spanish versions of the State and Trait Food Cravings Questionnaires. *Behav. Res. Ther.* **38**, 1125–1138 (2000).

2. Hallschmid, M., Wilhelm, I., Michel, C., Perras, B. & Born, J. A role for central nervous growth hormone-releasing hormone signaling in the consolidation of declarative memories. *PLoS One* **6**, e23435; 10.1371/journal.pone (2011).

3. Hummel, T., Kobal, G., Gudziol, H. & Mackay-Sim, A. Normative data for the “Sniffin’ Sticks” including tests of odor identification, odor discrimination, and olfactory thresholds: an upgrade based on a group of more than 3,000 subjects. *Eur. Arch. Otorhinolaryngol.* **264**, 237–243 (2007).

4. Steyer, R., Schwenkmezger, P., Notz, P. & Eid, M. *Der mehrdimensionale Befindlichkeitsfragebogen (MDBF). Handanweisung* (Hogrefe, 1997).

5. Tewes, U. *Hamburg-Wechsler-Intelligenztest für Erwachsene. Hawie R* (Huber, 1991).
